# Serum β-catenin changes vary among different stages of osteonecrosis of the femoral head: an exploratory biomarker study

**DOI:** 10.1186/s12891-022-05399-2

**Published:** 2022-05-10

**Authors:** Junyuan Huang, Yingchun Zhou, Wei Xiao, Peng Deng, Qiushi Wei, Weiguo Lu

**Affiliations:** 1 First Affiliated Hospital of Guangzhou University of Chinese Medicine, No. 16, Airport Road, Baiyun District, Guangzhou City, 510405 China; 2 Department of Pathogen Biology, Shenzhen University School of Medicine, Shenzhen, China; 3 Guangdong Research Institute for Orthopedics and Traumatology of Chinese Medicine, No. 16, Airport Road, Baiyun District, Guangzhou City, 510405 China; 4 Joint Center, The Third Affiliated Hospital of Guangzhou University of Chinese Medicine, Guangzhou, China

**Keywords:** Wnt pathways, β-catenin, ONFH

## Abstract

**Background:**

Wnt/β-catenin signaling pathway is closely related to the pathogenesis Osteonecrosis of the femoral head (ONFH). β-catenin, as a major component of Wnt signaling pathway, plays a vital role in the proliferation of osteoblasts. But the effect of altering β-catenin level on the early diagnosis and staging of ONFH has not been studied. Our purpose is to investigate the role of β-catenin level in the progress of ONFH.

**Method:**

One hundred and one patients with three stages of ONFH and fifty healthy controls were recruited between May 2016 and November 2016. We divided the patients into 32 cases of stage II, 41 cases of stage III and 28 cases of stage IV according to the Association Research Circulation Osseous (ARCO) classification. We evaluated the clinical bone histomorphology, expression position and level of β-catenin as well as the plasma β-catenin level. We investigated the level of β-catenin from the serum and tissue samples using ELISA and Western Blot assay. We also evaluated the expression of β-catenin in bone tissue by immunohistochemistry. Data were analyzed by independent t-test and ANOVA.

**Results:**

We found that the mean (± SD) serum level of β-catenin was 66.99 ± 3.032 ng/ml in the ONFH patients, which was higher than 20.14 ± 1.715 ng/ml observed in the control group (*P* < 0.001). Moreover, the β-catenin levels were 49.30 ± 4.649 ng/ml, 72.54 ± 4.864 ng/ml and 79.10 ± 4.773 ng/ml in the ONFH patients with ARCO stage II, stage III and stage IV respectively, showing significant difference among them (*P* < 0.001). We also found that the area under the curve (AUC) calculated by ROC curve analysis to determine the values for β-catenin levels in ONFH compared with those in the control group was 0.9358 (*P* < 0.001), where the sensitivity was 77.23% and specificity was 98.00%.

**Conclusion:**

Our results indicate that the increased β-catenin may play a vital role in the progress of ONFH and the level of β-catenin is correlated with ARCO stages. The cut-off concentration may be used as one of the sensitive marks to assess the disease process of ONFH.

**Supplementary Information:**

The online version contains supplementary material available at 10.1186/s12891-022-05399-2.

## Introduction

Osteonecrosis of the femoral head (ONFH) is a common orthopedic disease with high disability rate [1],  and this devastating disease is gradually becoming a global health problem. The etiology of ONFH includes traumatic and non-traumatic causes. It is well recognized that non-traumatic ONFH may associate with several risk factors including long-term steroid treatment, excess alcohol consumption, dysbarism, genetic mutation, smoking and autoimmune disease [2–6]. Although the exact mechanisms of ONFH currently remain in dispute, but the osteocyte apoptosis is known to be highly associated with osteonecrosis.

Wnt pathways are critical in regulating cell proliferation, apoptosis and differentiation, and they contribute to the pathologies of many diseases [7]. The canonical Wnt/β-catenin pathway is involved in regulating the process of bone formation and remodeling. β-catenin, as a major component of Wnt signaling pathway, plays a vital role in the proliferation of osteoblasts [8]. The expression of β-catenin is inhibited in ONFH. Suppressing β-catenin and Wnt signal-related molecular activities in osteoblasts and angiogenesis obviously reduces bone mass [9, 10]. And femoral head collapse always occurs after bone mass begins changing. But the effect of altering β-catenin level on progress of ONFH has not been studied. This study aims at investigating the level of β-catenin in patients with ONFH, comparing its difference among various stages of the disease, and revealing the potential relationship between it and disease progression.

## Materials and methods

### Study population and sample collection

Patients from the First Affiliated Hospital of Guangzhou University of Chinese Medicine who were diagnosed ONFH via medical history, physical examinations, X-ray and MRI between May 2016 and November 2016 were initially included. And then some of the patients with such concurrent conditions as congenital diseases, smoking, renal dysfunction, HIV infection, diabetes mellitus, cancer and cardiovascular disease were excluded. According to the ARCO staging system [11], the patients were divided into 32 cases of stage II, 41 cases of stage III and 28 cases of stage IV. Plasma was collected from the patients before the arthroplasty. 50 control plasmas were collected from healthy volunteers without hip pain nor any lesions showed in AP and frog-leg lateral pelvic radiographs, who received medical examination during the same period to the patients. ONFH bone sections (*n* = 18 totals, 3 cases of stage II, 4 cases of stage III and 11 cases of stage IV) were obtained after THA, while bone samples in the control group with femoral neck fracture (*n* = 6 totals) were collected. The weight-bearing zone in cartilage tissue was chosen for analysis. Bone samples were collected from healthy tissue or subchondral necrotic zone at 1-4 mm below the cartilage [12–14]. The general background of the ONFH patients and the healthy controls is shown in Table 1.

**Table 1:** General background

	ONFH	Stage II	Stage III	Stage IV	Controls	*P* value (ONFH VS Controls)
Total (n)	101	32	41	28	50	
Age(mean ± SD)	42.77 ± 14.646	40.03 ± 15.17	44.44 ± 13.20	43.46 ± 13.20	38.24 ± 16.08	> 0.05
Male	72	20	31	21	28	> 0.05
Female	29	12	10	7	22	
Bone (n)	18	3	4	11	6	
Age(mean ± SD)	43.67 ± 13.85	40.33 ± 14.74	39.5 ± 10.25	46.09 ± 15.31	40.33 ± 14.74	> 0.05
Male	13	2	3	8	4	> 0.05
Female	5	1	1	3	2	

### Bone morphology observed by H&E staining

Necrotic bone samples collected from necrotic area and healthy control femoral head were mounted in 4% formaldehyde for 24 h at room temperature. After subsequent decalcification with 10% EDTA over 2 weeks, the samples were embedded in paraffin wax. Specimens cut longitudinally into 5-μm sections were stained with hematoxylin–eosin(H&E). The stained sections were observed through the microscope (BX53, Olympus Corp., Japan) [12–14].

### Immunohistochemistry for β-catenin

Immunohistochemistry was used to further analyze the expression of protein β-catenin. The femoral head tissue sections obtained from previous sections/2.2 were processed with avidin–biotin-peroxidase complex (ABC) to detect the presence of activated β-catenin. After being deparaffinized in xylene and rehydrated by a graded series of alcohols to water, and then incubated in 0.3% H_2_O_2_ for 1 h to quench the endogenous peroxidase activity, the sections were incubated with primary rabbit anti-human β-catenin antibody (1:200; Santa Cruz Biotechnology, USA) in a solution consisting of 1% bovine serum albumin and 0.05% sodium azide in 0.1 M PBS for 24 h at 4 °C. After three washes with PBS, the specimens were exposed to biotinylated goat anti-rabbit IgG diluted 1:200 in PBS for 4 h at room temperature. Next, the peroxidase reaction was developed for 10 min in 0.05 M Tris buffer (pH 7.6). Slides were covered with DEPEX mounting medium, and observed under a microscope (BX53, Olympus) [12–14].

### Western blotting for β-catenin

The bone samples were washed with 0.9% NaCl and PBS and lysed by NET-Triton lysis buffer. Aliquots of lysates were electrophoresed on SDS-PAGE with Tris–glycine running buffer and then the proteins were transferred to poly membranes (BioRad). Nonspecific binding of the antibodies to the membrane was blocked by a one hour incubation with TBS/Tween20 (0.05 mM Tris, 0.15 mM NaCl, pH 7.6; 1% Tween 20) containing 5% w/v non-fat dried milk for 1 h and were then incubated in TBS/Tween20 with 5% w/v non-fat dried milk supplemented with specific rabbit anti-human β-catenin antibody (1:1000; Santa Cruz Biotechnology, USA) overnight at 4 °C. Horseradish peroxidase-conjugated anti-β-actin (Santa Cruz Biotechnology, USA) was used as loading control. Signals were detected using a diluted (1:5000) secondary polyclonal antibody (goat anti-rabbit conjugated with peroxidase) and the membranes were immersed in ECL detection solution (Santa Cruz, USA). The protein bands were quantified using an Epson GT-8000 laser scanner [12–14].

### β-catenin level measurements by Enzyme-linked immunosorbent assay

Levels of β-catenin in plasma were analyzed by a commercial sandwich enzyme-linked immunosorbent assay (ELISA) (IBL, Germany). The concentration of β-catenin in the samples was determined by comparing the O.D. of the samples to the standard curve. ROC curves were used to draw data from the results obtained in this study, and the cut-off value was set to provide optimal diagnostic accuracy and likelihood ratios for the level of β-catenin [12–14].

### Statistical analysis

Statistical analysis was performed using SPSS 23.0 and GraphPad Prism v7.0. Unpaired t-tests with the significance level of *P* < 0.05 were used for statistical analysis.

## Result

### Radiography and pathology evaluation of ONFH patients and control subjects

**Fig. 1:**
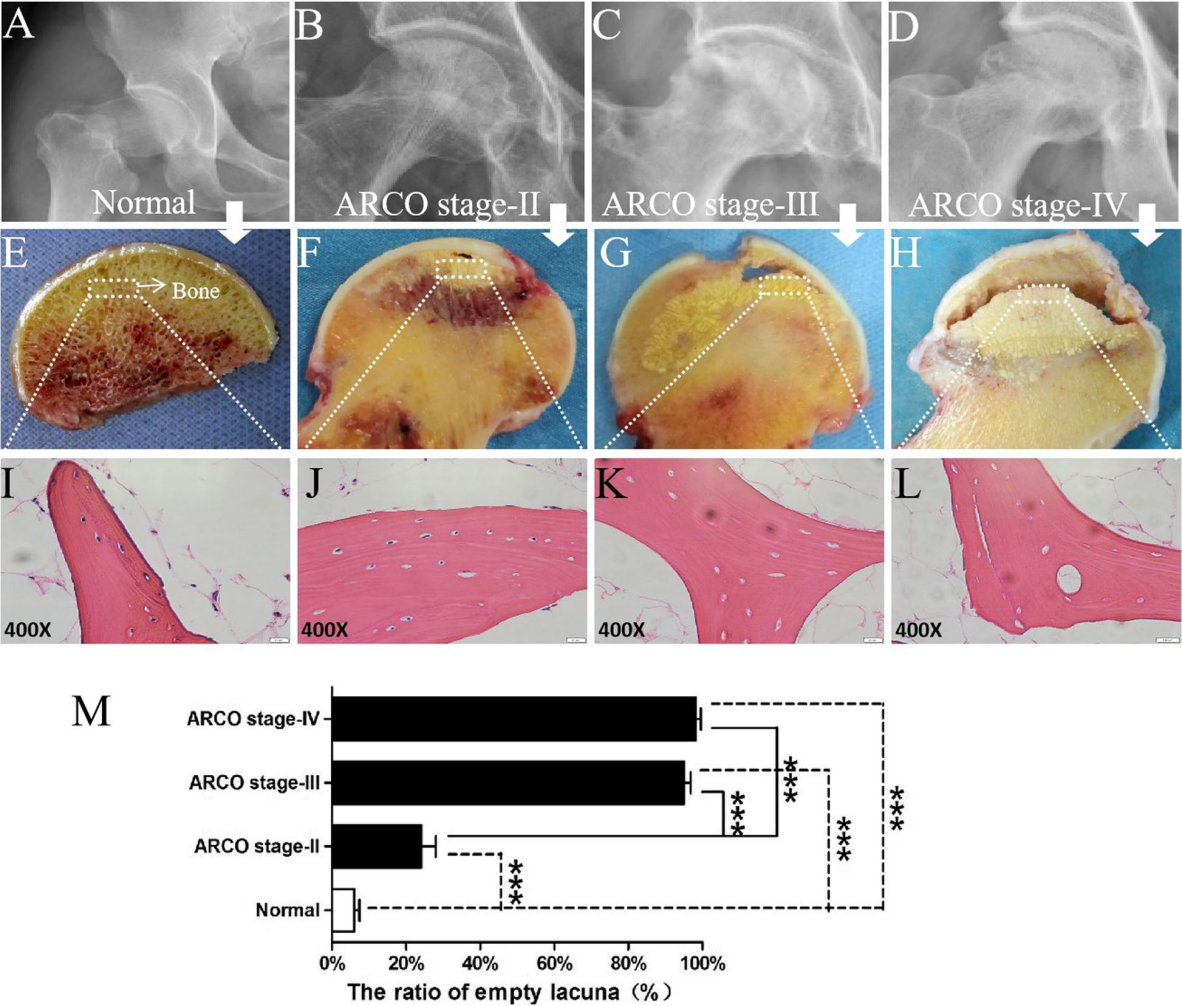
**A-D** X-ray images of control subject and ONFH patients with different ARCO stage. **E–H** General appearance in the bone and cartilage samples of control subject and ONFH patients with different ARCO stage. The white dashed boxes indicated the regions collected for further analysis. **I-L** Histopathological features of control and ONFH bone. **M** The ratio of empty lacunae in each ONFH groups of different stage and control group. ^***^P < 0.001

Figure 1A-D show the X-ray results from the normal control group and the ONFH patients. Figure 1A shows a normal joint space in the femoral head, which also has a regular shape and homogeneous spherical density. Figure 1B shows heterogeneous densities, disappearance of local bone trabeculae, and a normal joint space corresponding to ARCO stage II. Figure 1C shows the collapse of the articular surface, osteosclerosis and the preservation of joint space, indicating a sign of stage III. Figure 1D shows acetabulum changes, subchondral collapse and degenerative arthritis. Figure 1E-H show the general appearance of femoral head sections. Figure 1E shows the homologous trabecular bone of the control subject without any evident destruction. Figure 1F shows the disorganized bone trabeculae and rough surface cartilage in the necrotic region. Figure 1G and 1H show the distinct collapse of the femoral head. Furthermore, the deteriorated and severely destroyed, even obviously stripped in the cartilage structure are shown in Fig. 1H. Images from HE staining are shown in Fig. 1I-L. Figure 1I shows a healthy and complete trabecular bone structure with lots of osteocytes embedded in the control bone samples. Figure 1J-L show that with a higher severity of the stage, the bone trabeculae displayed an increasing empty lacunae resulting from the loss of osteocytes. Figure 1M shows that the ratio of empty lacunae in the control group was significantly lower than those in each ONFH groups of different stages (*P* < 0.001). Although no differences were found between stage III and stage IV (*P* > 0.05), the ratio of empty lacunae in both stages III and IV were higher than that in stage II (*P* < 0.001).

### Immunohistochemistry for β-catenin

**Fig. 2:**
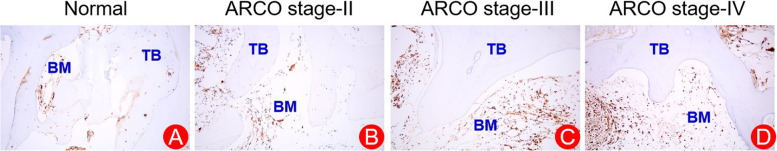
Immunohistochemistry results for β-catenin of cartilage samples in control group (*n* = 1) and ONFH group with different stages. Each stage contains one sample

Both necrotic femoral head region and healthy region of the femoral head positive for β-catenin were evaluated by immunohistochemical staining (Fig. 2). Intact femoral head and low-level presence of β-catenin were observed in healthy samples (Fig. 2A). The level of β-catenin was increased among those with ARCO stage progress and was accompanied by a rise of empty bone lacuna (Fig. 2B-D). Furthermore, with the progression of disease, the superficial layer of femoral head had become rough, disordered and even structurally disappeared in the control group and the ONFH groups with different stages.

### Western blot quantitative analysis of ONFH patients and control subjects

**Fig. 3:**
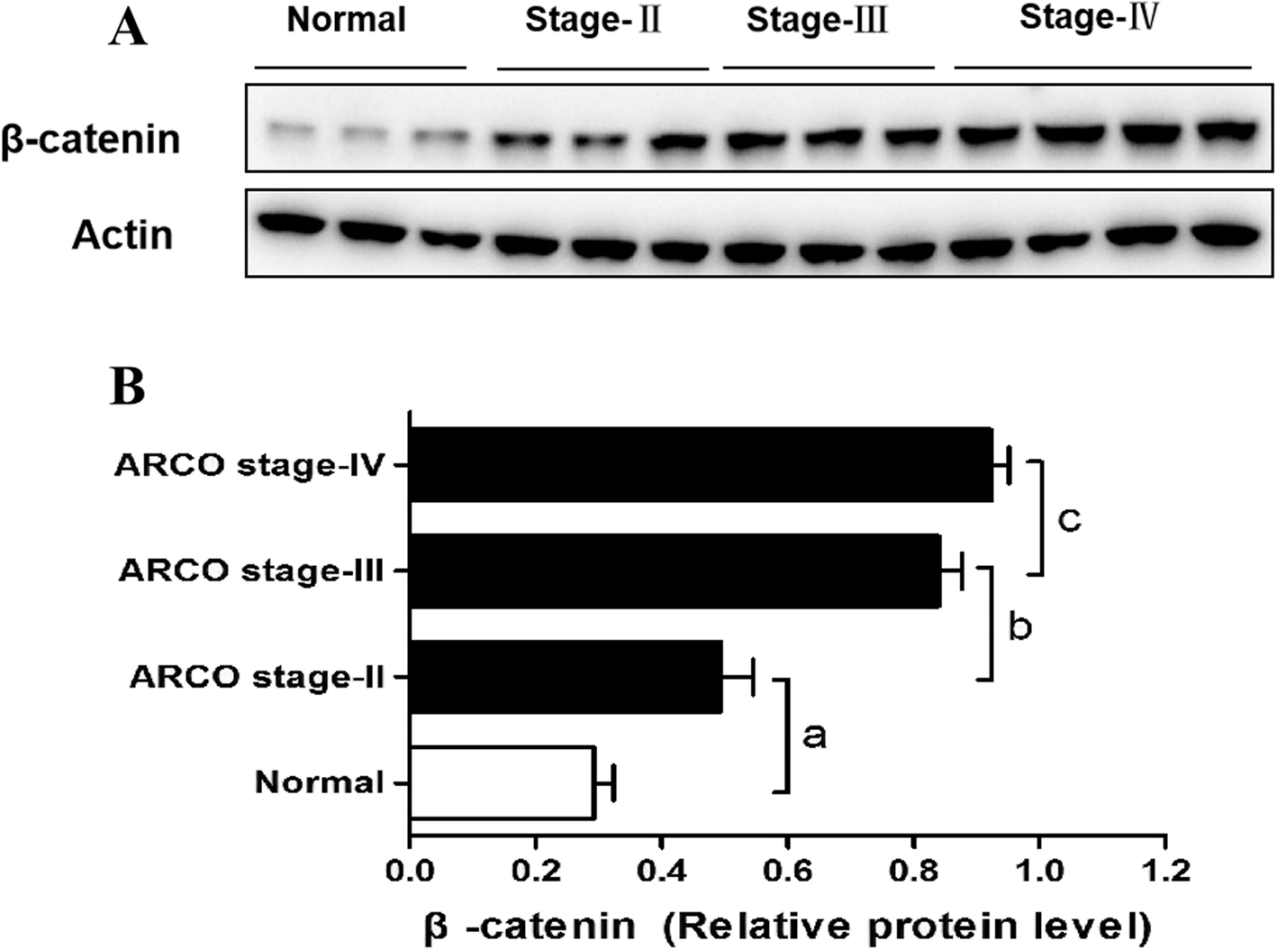
Western blot analysis of cartilage samples for the β-catenin. **A** Level of β-catenin is increased with the ARCO stage. 3 samples in control, 3 samples in stage II, 3 samples in stage III, and 4 samples in stage IV were done. **B** The histogram represents western blotting analysis. Values are the means ± SEM. ^a^*P* < 0.001 vs the control group; ^b^*P* < 0.001 vs the stage II group; ^c^*P* < 0.001 vs the stage III group

The expression of β-catenin was determined by Western blotting (Fig. 3). The level of β-catenin in femoral head of ONFH patients was significantly higher than those in control group (*P* < 0.001). Among the three ARCO stages, the level of β-catenin in the ONFH patients increased with the severity of X-ray findings increased.

#### Plasma β-catenin level quantity of ONFH patients and control subjects

**Fig. 4:**
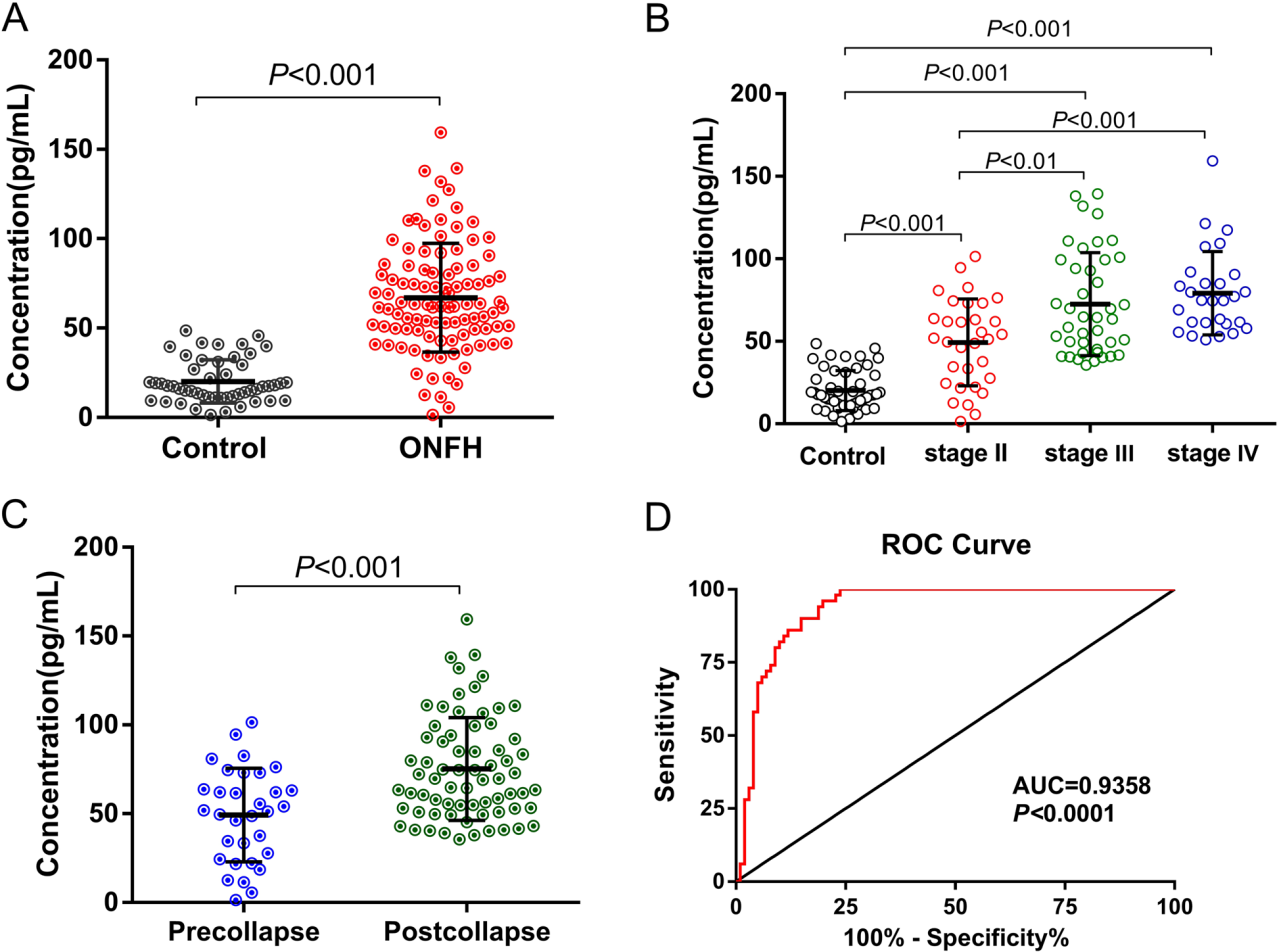
Overview shows plasma β-catenin level in ONFH patients and control individuals. **A-C** β-catenin levels with statistical differences among different groups. **D** Receiver operating characteristic (ROC) curve and the area under the curve (AUC) in association with the sensitivity and specificity of ONFH

The result of plasma β-catenin level measured by ELISA and potential relation between other clinical data was shown in Table 2. The mean (± SD) serum level of β-catenin was 66.99 ± 3.032 ng/ml in the ONFH patients, which was higher than 20.14 ± 1.715 ng/ml observed in the control group ( t = 10.46, *P* < 0.001) (Fig. 4A). Moreover, the β-catenin levels were 49.30 ± 4.649 ng/ml, 72.54 ± 4.864 ng/ml and 79.10 ± 4.773 ng/ml in the ONFH patients with ARCO stage II, stage III and stage IV respectively, showing significant difference among them (DF = 3, *F* = 51.39, *P* < 0.001). According to multiple comparisons, difference of plasma β-catenin level was found between stage II and stage III (t = 4.105, *P* < 0.01), and between stage II and stage IV (t = 4.798, *P* < 0.001) (Fig. 4B). In addition, β-catenin level in post-collapse patients (75.20 ± 3.478 ng/ml) was higher than that in pre-collapse patients (49.30 ± 4.649 ng/ml) (t = 4.497, *P* < 0.001) (Fig. 4C). The area under the curve (AUC) which was calculated by ROC curve analysis to determine the values for β-catenin levels in ONFH compared with those in the control group was 0.9358 (*P* < 0.0001), where the sensitivity was 77.23% and specificity was 98.00% (cut-off, 45.99 ng/ml) (Fig. 4D).

**Table 2:** Plasma β-catenin levels in ONFH patients and control subject and potential relation between other clinical data

Groups	Cases	β-catenin level (ng/ml)	Comparison	*P* value
Control	50	20.14 ± 1.715	Control vs ONFH	< 0.001
ONFH	101	66.99 ± 3.032		
Pre-collapse	32	49.30 ± 4.649	Pre-collapse vs Post-collapse	< 0.001
Post-collapse	69	75.20 ± 3.478		
ARCO stages				< 0.001
Stage II	32	49.30 ± 4.649	II vs III	< 0.01
Stage III	41	72.54 ± 4.864	III vs IV	> 0.05
Stage IV	28	79.10 ± 4.773	II vs IV	< 0.001
Etiology				0.0821
Alcohol-induced (a)	41	71.61 ± 4.397		
Steroid-induced (b)	32	55.57 ± 5.988		
Idiopathic (c)	12	72.85 ± 6.443		
Traumatic (d)	16	73.61 ± 7.618		

Data presented as mean ± SD

## Discussion

In the present study, we investigated the relationship between β-catenin level and disease process in the patients with ONFH. We found that at both protein level and plasma level, β-catenin was significantly higher in the ONFH group, especially in the post-collapse patients than those in the control group, and it was positively associated with ARCO stages, but not with etiology. To the best of our knowledge, this is the first study effectively explaining the correlation between β-catenin level and the disease severity of ONFH. Our finding suggests that β-catenin could possibly be used as a biomarker to assess the progress of ONFH.

Wnt/β-catenin signaling pathway which regulates the differentiation of bone marrow mesenchymal stem cells is closely related to the regulation of bone balance [15]. β-catenin mainly exists in the cytoplasm, but a few of them also exists in the cell membrane and the nucleus. And as the core target and important regulator, it plays a crucial role in Wnt/β-catenin pathway. In the normal Wnt/β-catenin signaling pathway, β-catenin will pass from the cytoplasm into the nucleus for promoting the proliferation and differentiation of osteoblast, and keeping bone balance. On the contrary, when Wnt signaling pathways is suppressed, β-catenin will be degraded in the cytoplasm, thus weakening the proliferation and differentiation of osteoblast, causing a decline in bone mass [16]. However, many studies in recent years have shown that Wnt/β-catenin signaling pathway has different effects on osteoblasts of different differentiation stages, and it needs precise regulation to maintain bone balance [17, 18]. At present, numerous studies have indicated that ONFH is related to osteocyte apoptosis and bone remodeling, with increased osteocyte apoptosis and bone resorption in the necrotic area of the femoral head, but increased osteoblast activity in the sclerotic area around the necrotic area [19–21], these results are consistent with the phenomenon we found in the present study. According to the results of immunohistochemistry and plasma, our research also showed that the expression of β-catenin in ONFH patients was higher than that in normal control group, and it was positively associated with ARCO stages. At the early stage of ONFH, corresponding to ARCO I, bone cell apoptosis was the main manifestation of femoral head lesions. Therefore, the expression of β-catenin might be decreased. Because most patients at the early stage of ONFH are asymptomatic and hardly found in medical institutions, our research did not include any of them, however we will further study this subgroup. However, sclerosis rim can be found in X-ray film at the stage of ARCO II, which mean the osteoblast activity was enhanced. Thus, in theory, as the disease progresses, the osteoblast activity increases and so does the expression level of β-catenin. Although some animal experiments have shown that the expression of β-catenin in rats with femoral head necrosis was decreased [22–24], but this is not contradictory, because these animal experiments only reflect the situation of early stage of ONFH.

In addition, the results from the X-ray film, observations of the femoral head section as well as the HE staining showed that the pathological features of ONFH are apoptosis of osteocytes and rising number of lacunae. The ratio of lacunae was increased as the disease proceeded, confirming that the samples collected are trustworthy and capable of proving the pathological alterations in different stages. Although the exact mechanisms of ONFH are now still in dispute, but the femoral head cartilage damage caused by osteocyte apoptosis is seen highly associated with osteonecrosis [25].

There are certain limitations in this study. First, we have not evaluated the levels of β-catenin in stage I, due to the difficulty to enroll patients at an early stage of ONFH. Second, a relatively small sample size has limited the accuracy of the research. Even with these limitations, our study is the first one demonstrating that β-catenin could possibly be used as a marker for disease progression of ONFH.

## Conclusion

Our findings indicate that the increased β-catenin may play a vital role in the progress of ONFH and the level of β-catenin is correlated with ARCO stages. The cut-off concentration may be used as one of the sensitive markers to assess the disease process of ONFH.

## Supplementary Information

**Additional file 1.**

## Data Availability

All data generated or analyzed during this study are included in this published article.

## Abbreviations

ONFH: Osteonecrosis of the femoral head
ARCO: Association Research Circulation Osseous
ELISA: Enzyme-linked immunosorbent assay
AUC: The area under the curve
