# Supplementary material for: Serum β-catenin changes vary among different stages of osteonecrosis of the femoral head: an exploratory biomarker study
Source: BMC Musculoskelet Disord. 2022 May 10;23:434. doi: 10.1186/s12891-022-05399-2 (PMC9088107; doi:10.1186/s12891-022-05399-2)
Supplement: Supplementary file 1 — Additional file 1. [file 12891_2022_5399_MOESM1_ESM.pdf]

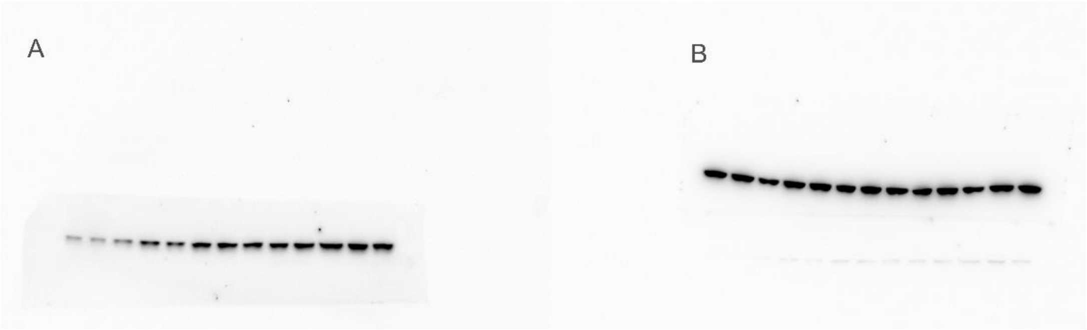

Supplemental figure 1. The original unprocessed Western blot images of figure 3A. **A** Western blot of β-catenin in figure 3A. **B** Western blot of actin in figure 3A.
